# Portable and Point-of-Care Testing Approach for Determining Soil Extracellular Enzyme Activities

**DOI:** 10.3390/mi17050599

**Published:** 2026-05-14

**Authors:** Xu Han, Fangzhou Zhang, Ruirui Chen, Weixin Wang, Yongjie Yu, Zaijiong Yi, Jingyi Yang, Bo Liu, Shilun Feng, Jun Li, Youzhi Feng

**Affiliations:** 1 State Key Laboratory for Development and Utilization of Forest Food Resources, Nanjing Forestry University, Nanjing 210037, China; hanxu@njfu.edu.cn (X.H.); rrchen@njfu.edu.cn (R.C.); 2 Xiangfu Laboratory, Jiashan 314102, China; fang123zhuan@163.com (F.Z.); liubo@xflab.org.cn (B.L.); shilun.feng@mail.sim.ac.cn (S.F.); 3 Shanghai International Travel Healthcare Center, Shanghai Customs District, Shanghai 200335, China; vivianweiwei5@sina.com (W.W.); 23111010089@m.fudan.edu.cn (Z.Y.); 4 Jiangsu Collaborative Innovation Center for Solid Organic Waste Resource Utilization, Nanjing 210095, China; 5 School of Materials Engineering, Changzhou Vocational Institute of Industry Technology, Changzhou 213164, China; yu.yongjie@hotmail.com; 6 Nanjing Institute of Geology and Palaeontology, Chinese Academy of Sciences, Nanjing 210008, China; jyyang@nigpas.ac.cn; 7 State Key Laboratory of Transducer Technology, Shanghai Institute of Microsystem and Information Technology, Chinese Academy of Sciences, Shanghai 200050, China

**Keywords:** centrifugal microfluidics, soil extracellular enzymes, soil health assessment, fluorescence detection, enzyme activity quantification

## Abstract

Soil eco-enzymes (i.e., microbial extracellular enzymes) play essential roles in terrestrial nutrient cycling and support ecosystem services. In this regard, their activities serve as indicators of soil health. However, conventional spectrophotometric and microplate fluorometric assays are often limited by lengthy reaction procedures, relatively high reagent consumption, and insufficient compatibility with complex soil matrices. In this investigation, we developed a portable, centrifugally driven microfluidic chip for the rapid and sensitive determination of multiple soil extracellular enzyme activities. This integrated platform automated sample aliquoting, reagent metering, mixing, and sedimentation, enabling the parallel measurement of eight enzymes. Such system demonstrated precise liquid control via capillary valves and high optical uniformity (<5% fluorescence variation). 4-methylumbelliferone (MUF)-based calibration exhibited strong linearity (R^2^ > 0.99) across diverse soil types. Compared with conventional microplate assays, the microfluidic method improved reproducibility (CV < 15%), enhanced the detection of weak fluorescence signals, and increased throughput while reducing reagent consumption. This field-ready platform provides a robust solution for standardized soil enzyme assessment and offers future potential for integration with AI-driven data analytics and large-scale ecological monitoring frameworks.

## 1. Introduction

Soil extracellular enzymes, secreted by microorganisms (e.g., bacteria and fungi) and plant roots into the surrounding matrix, are critical catalysts in soil ecosystems, driving material cycles and energy flows [1,2]. By depolymerizing complex organic matter, they mediate the transformation and reutilization of C, N, and P, thereby influencing soil structure and fertility [3].

Different enzymes exhibit distinct substrate preferences and ecological functions. For example, β-glucosidase (BG) hydrolyzes β-1,4-linked polysaccharides such as cellulose and cellobiose to release glucose as an accessible carbon source for microbes [4,5]; β-xylosidase (BX) degrades xylan chains in hemicellulose to release xylose and accelerate plant residue decomposition [6]; N-acetyl-β-glucosaminidase (NAG) primarily targets N-bearing organics such as chitin and glycoproteins, cleaving N-acetyl-β-glucosaminide bonds to release amino acids and ammonia (NH_3_) as nitrogen sources [7]; Phosphatases (AP) hydrolyze phosphate monoesters in organic matter, mineralizing organic P into inorganic phosphate (e.g., H_2_PO_4_^−^, HPO_4_^2−^) and thus sustaining P cycling [8,9]. In addition, certain oxidoreductases and hydrolases participate in the transformation and mineralization of organic pollutants, shaping their environmental behavior and fate [10]. Different extracellular enzymes exhibit a clear division of labor, each catalyzing distinct stages of biochemical reactions. These enzymes also engage in synergistic interactions, which collectively sustain ecosystem stability [11]. Soil extracellular enzyme activities (EEAs) not only reflect nutrient transformation efficiency and overall soil health, but also reveal the potential for pollutant degradation and the functional status of ecosystems. Therefore, accurate quantification of soil EEAs is crucial for elucidating soil ecological processes, assessing soil health, and guiding agricultural practice [12].

A wide array of methods is currently used worldwide to assess soil EEAs, including spectrophotometry, microplate fluorometry, isotope labeling, and the titrimetric procedures employed in early studies [13]. Among these approaches, spectrophotometric methods are highly susceptible in their sensitivity to interference from colored soil constituents, such as humic substances, which render quantitative analysis of low-activity samples unreliable [14]. Although isotope labeling techniques offer high specificity and traceability, they require complex sample pretreatment and are prohibitively expensive, while traditional titrimetric analyses have largely been phased out because of their insufficient accuracy and reproducibility [15]. Consequently, microplate fluorometric assays employing fluorescent substrates such as 4-methylumbelliferone have become widely used in practice to improve detection sensitivity and throughput [16]. This approach lowers the limit of detection and enables multiple reactions to be carried out in parallel, but it also has several obvious drawbacks: fluorescent substrates and microplate readers are costly [17], and the workflow involves multiple manual steps, including dispensing, mixing, incubation, and signal acquisition, so the entire assay is overly dependent on manual operation and centralized laboratory equipment [18]. These factors restrict its routine use in resource-limited laboratories, regional monitoring stations, and field observatories lacking dedicated plate readers. Moreover, in practical applications, different laboratories frequently adjust their protocols according to soil type, instrumentation, and operator habits, leading to subtle differences in parameters such as substrate concentration and reaction time. Such deviations may introduce systematic biases in the measured enzyme activities and ultimately compromise the comparability of soil enzyme activity datasets across studies [19]. In summary, conventional methods struggle to balance speed, accuracy, portability, and multiplexing capacity, and fall short of meeting the comprehensive needs for extracellular enzyme activity measurements in contemporary soil health assessment and ecosystem process research. Therefore, there is an urgent need to develop a portable and integrated analytical platform that can miniaturize and automate the entire assay workflow, standardize reaction conditions across laboratories, reduce reagent consumption and operator dependence, and enable high-throughput, multiplexed determination of soil extracellular enzyme activities in both laboratory and field settings.

In recent years, microfluidics has emerged as a novel analytical platform that, leveraging micrometer-scale channel networks and fluid manipulation at the micro and nanoscale, enables sample separation, reaction, and detection within minute volumes [20,21]. Through integration with complementary technologies such as optical, electrochemical, and biosensing modalities, microfluidic platforms can further expand their analytical capabilities [22,23]. They have shown broad application potential in biomedical research, chemical analysis, and environmental monitoring [24,25,26,27]. The integration of sample metering, reaction, separation, and signal readout into a single miniaturized platform has been demonstrated in various 3D-printed microfluidic devices for diverse targets, including heavy metal ions, formaldehyde, norovirus, and *E. coli* [28,29,30,31]. These studies highlight the broad analytical potential of microfluidic platforms and their key advantages, by constructing channels, valves, and pumps on a substrate, microfluidic systems integrate complex experimental workflows into continuous, automated operations, offering advantages of high throughput, low reagent consumption, cost-effectiveness, operational simplicity, and high sensitivity [32,33]. Such microfluidic systems directly address key drawbacks of conventional microplate fluorescence assays, including labor-intensive workflows, high instrument costs, and strong dependence on centralized laboratories. They thus provide a promising route toward portable and standardized enzyme activity measurements. Nevertheless, practical applications in complex soil matrices remain nascent, and systematic investigations specifically targeting soil extracellular enzyme activities are particularly limited [34]. To better clarify the contribution of the present method relative to previous soil-related microfluidic platforms, representative studies are compared in Supplementary Table S1 in terms of detection principle, readout method, target analyte, relative detection cost, and portability.

**Figure 1:**
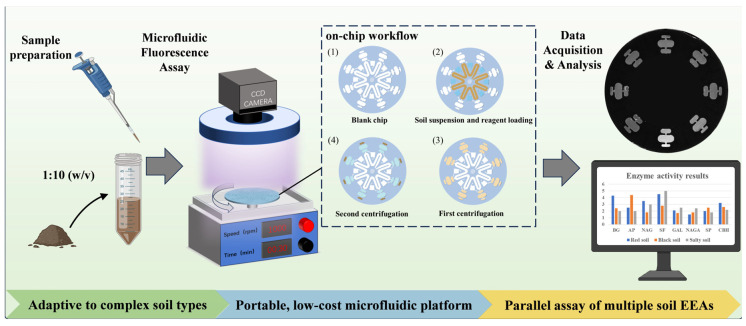
Schematic diagram of the assay workflow, including preparation of soil suspensions at a 1:10 (*w*/*v*) dilution, microfluidic fluorescence detection with a CCD camera, violet ring light, and centrifugal platform, the on-chip mixing of soil samples, and representative fluorescence images with subsequent data processing.

To address the methodological limitations outlined above, we present an integrated microfluidic strategy that couples fluorogenic-substrate assays with a centrifugally driven, parallel-architecture chip for the multiplexed quantification of soil extracellular enzyme activities (Figure 1). By consolidating the entire workflow from soil suspension injection through on-chip mixing to fluorescence readout within a single, lightweight device. The method eliminates the need for bulky plate readers, multi-step pipetting, and dedicated laboratory space, thereby overcoming both the throughput limitations and the portability constraints that have long restricted conventional microplate assays for soil enzyme analyses. This work not only establishes a robust, field-deployable platform for standardized enzyme activity measurement in complex soil matrices but also provides a scalable analytical framework for future ecological monitoring and soil health assessment.

## 2. Materials and Methods

### 2.1. Materials and Instruments

For the target extracellular enzymes, derivatives of 4-methylumbelliferone (MUF) were selected as specific fluorescent substrates, and MES buffer (10 mM, pH 6.1) was used as the reaction buffer system, both purchased from Sigma-Aldrich, St. Louis, MO, USA. The names of the substrates and the corresponding extracellular enzymes to be measured in the soil samples are listed in Table 1. The preparation method for the substrates is as follows: an appropriate amount of substrate was weighed and dissolved in 1 mL of methoxyethanol, then diluted with water to a final volume of 5 mL to obtain a 10 mM stock solution. The substrate stock solution was stored at −20 °C in the dark to maintain its stability. The chip fabrication material used was polydimethylsiloxane (PDMS), purchased from Dow Corning, Freeland, MI, USA. The chip molds were fabricated by Hua Jing Yong Sheng Mechanical Technology Co., Ltd. (Dongguan, China). The UV light source used in the experiments was purchased from Shenzhen Boyiyu Technology Co., Ltd. (Shenzhen, China), and the optical filter was purchased from Wanji Instruments (Beijing, China). A CCD camera (model LBAS-U3250-14C) provided by Xiangfu Laboratory (Jiashan, China) was used for imaging.

### 2.2. Design and Fabrication of the Chip

The centrifugal microfluidic chip was designed as a radially symmetric disk containing eight independent detection units for parallel enzyme assays. Each detection unit consisted of a soil chamber, reagent chamber, overflow chamber, reaction chamber, and sedimentation chamber. The soil chamber was used for loading the soil suspension, while the reagent chamber was used for preloading the fluorogenic substrate solution and reaction buffer. The overflow chamber was designed to accommodate minor volume deviations during liquid loading, and the sedimentation chamber was used to collect soil particles after centrifugation, thereby reducing optical interference during fluorescence detection.

**Figure 2:**
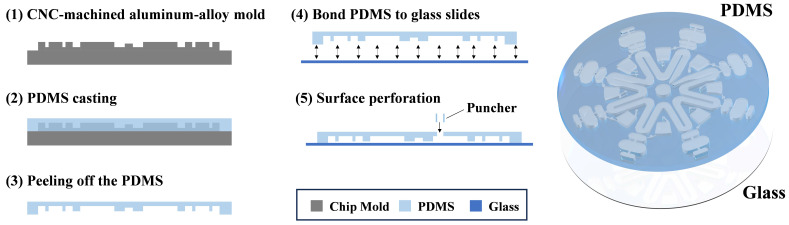
Fabrication workflow of the microfluidic chip.

The chip adopts a bilayer PDMS–glass architecture to balance optical transparency and mechanical stability. The fabrication workflow was as follows (Figure 2): First, a three-dimensional model was designed in SolidWorks 2024, and an aluminum mold was machined by CNC. PDMS prepolymer and curing agent (10:1, *w*/*w*) were thoroughly mixed, degassed in a vacuum desiccator for 10–15 min, and uniformly cast into the mold. After curing at 80 °C for 2–3 h and cooling, the PDMS replica bearing the microchannel structures was demolded. Inlet and vent holes (1 mm diameter) were punched at predefined locations. The PDMS layer and glass substrate were then treated in an oxygen plasma (720 V, 0.230 A, 50 s) for surface activation, immediately aligned, and bonded. The bonded chips were thermally annealed at 70–80 °C for 30–40 min to promote silanol condensation and formation of Si–O–Si covalent bonds, thereby enhancing bond strength and sealing performance [35]. Prior to use, all chips underwent visual inspection and channel-continuity tests to ensure the absence of bubbles, leakage, and clogging.

### 2.3. Working Principle of the Microfluidic Fluorescence Assay

**Figure 3:**
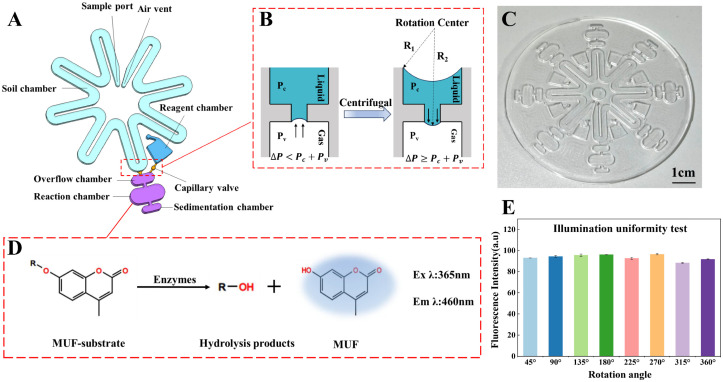
Microfluidic Chip Design and Testing. (**A**) Functional schematic of the microfluidic-chip chambers; (**B**) Principle of capillary-valve-mediated liquid control; (**C**) Photograph of the fabricated microfluidic chip; (**D**) Schematic of the MUF-based assay principle; (**E**) Fluorescence signals from the same Reaction chamber at different rotation angles.

The assay principle was based on the enzymatic hydrolysis of fluorogenic substrates. Each substrate consisted of an enzyme-specific cleavable moiety, denoted as “R”, and a fluorescent reporter, MUF. In the intact substrate, the fluorescence of MUF was largely quenched and therefore weakly emissive. Upon hydrolysis by the corresponding extracellular enzyme in the soil suspension, the substrate released free MUF, which produced a fluorescence signal with excitation at 365 nm and emission at 460 nm (Figure 3D). The fluorescence intensity was subsequently converted to MUF concentration using the corresponding calibration curve and further used to calculate extracellular enzyme activity.

### 2.4. Experimental Design and Soil Samples

Three sets of experiments were conducted to evaluate the performance of the microfluidic chip and to demonstrate its applicability to soils with different properties. (i) Construction of soil-specific MUF calibration curves: Fresh fluvo-aquic, red, black, and saline-alkali soils were used to construct soil-specific MUF calibration curves. Standards were prepared in the same matrix as the samples: using a 5 mM MUF stock, 100 μL soil suspension was added to each reaction unit and combined with MES buffer and appropriate volumes of the stock to a final volume of 150 μL, yielding 0, 3, 6, 9, and 12 μmol·L^−1^. Standards and samples underwent identical spin, incubation, and detection procedures. (ii) Determination of eight extracellular enzyme activities on the microfluidic chip: For each soil type, MES buffer, MUF-labelled substrates and soil suspensions were loaded onto the chip, which was then subjected to a unified sequence of centrifugation, incubation and fluorescence detection. Fluorescence signals were converted to activities of eight enzymes (BG, CBH, BX, GAL, NAG, NAGA, SF and AP) using the corresponding calibration curves. Eight technical replicates per enzyme were obtained using eight independently prepared microfluidic chips, each enabling simultaneous measurement of all eight enzymes. (iii) Method comparison in saline–alkali soils: Mildly and severely saline–alkali soils were analyzed in parallel by the microfluidic chip and a conventional 96-well microplate fluorometric assay. The two methods used the same buffer system, substrate concentrations and incubation conditions, allowing comparison of detection precision and experimental efficiency.

All soils were sieved through a 2 mm stainless steel mesh to remove roots, stones, and other debris, which effectively prevented clogging of the microfluidic chip channels during subsequent analysis and ensured stable system operation. The sieving was performed using fresh soil without air-drying to prevent the loss of moisture, which could lead to a reduction in extracellular enzyme activity.

### 2.5. Determination of Soil Extracellular Enzyme Activities

During enzymatic activity determination, 10 mM MES buffer (pH 6.1) is adopted as the reaction medium. Working solutions of each substrate were prepared in water at a concentration of 400 μM. Prior to assay initiation, preloading was performed manually using a micropipette. Specifically, 25 μL of MES buffer and 25 μL of the corresponding substrate working solution were premixed and introduced into each reagent chamber, after which the inlet was sealed with transparent film to prevent leakage and evaporation. Soil suspensions were prepared at a mass-to-volume ratio of 1:10 (1 g soil to 10 mL solvent, *w*/*v*); after thorough dispersion by vortexing or brief ultrasonication, 800 μL of the soil suspension was immediately loaded into the sample chamber and sealed in the same manner. After both the reagent and sample had been loaded, the chip was mounted on a spin coater-based centrifugal platform beneath a ring UV light source and a CCD camera. An initial spin at 1000–1500 rpm for 6–10 s drove the soil suspension and reagents simultaneously into, and to fill, the reaction chambers. Subsequently, chips were incubated at 30 ± 3 °C in the dark for 30 min. To terminate the reaction and clarify the supernatant, a subsequent centrifugation step was conducted at 3500–4500 rpm for 20–30 s to transport soil particles into the sedimentation chamber. Fluorescence signals from each reaction chamber were finally recorded under the conditions of an excitation wavelength (λex) of 365 nm and an emission wavelength (λem) of 460 nm. For each sample, three parallel channels were used. A blank control (with deionized water substituting for the soil suspension) was included, and a calibration curve was constructed. After blank subtraction, fluorescence signals were converted to product concentrations using the standard curve.

### 2.6. Quantitative Analysis of Fluorescence Images

Fluorescence images obtained from the microfluidic chip were analyzed using ImageJ (version 1.54p). All images were first converted to 16-bit grayscale format. Identical regions of interest (ROIs) were defined within each reaction chamber while avoiding chamber edges, bubbles, and sedimented particles. For all data obtained using the microfluidic chip method, the raw fluorescence intensity was extracted as the mean gray value of each ROI in ImageJ. Signals from parallel channels of the same sample were averaged to obtain the sample value. Background was removed by subtracting the signal from the corresponding ROI in the blank control, yielding net fluorescence intensities. Net values were then converted to product concentrations using the MUF standard curve for the corresponding substrate, from which enzyme activities were calculated. Data curation and plotting were performed in Microsoft Excel 2016 and OriginPro 2024.

## 3. Results and Discussion

### 3.1. Microfluidic Chip Design and Optimization

To evaluate the performance of the centrifugal microfluidic chip described in Section 2.2, we further characterized its structural design, liquid-control behavior, and optical detection compatibility. The specific chamber configuration of the designed chip is schematically shown in Figure 3A. The chip was fabricated as a radially symmetric disk with a radius of approximately 40 mm and a PDMS-layer thickness of 3–4 mm. The microchannels were approximately 0.5 mm in both width and height, providing stable flow while maintaining sensitive actuation of the capillary burst valves. The V-shaped through sample chamber had a volume of (800 ± 10) μL, while the reagent chamber, overflow chamber, and combined reaction–sedimentation chamber had volumes of (50 ± 2) μL, (20 ± 3) μL, and (140 ± 5) μL, respectively. In total, the chip contained eight independent and parallel detection units, enabling simultaneous multi-enzyme assays within a single disk.

The chamber layout was optimized to improve liquid distribution, reaction stability, and fluorescence readout in complex soil suspensions. In each detection unit, the overflow chamber was incorporated to buffer small loading-volume deviations and maintain uniform liquid aliquoting among parallel channels. The sedimentation chamber was positioned downstream of the reaction chamber to collect suspended soil particles after the second centrifugation step, thereby reducing particle-induced light scattering and background interference in the fluorescence detection region. This optimized configuration enables sample loading, reagent metering, on-chip reaction, particle clarification, and optical detection to be integrated within each radial unit, improving the operational stability and reproducibility of the multiplexed assay.

Following this structural optimization, to achieve precise aliquoting control, geometric capillary valves were incorporated at key junctions along each branch. The liquid-control principle is illustrated in Figure 3B. At rest, interfacial tension prevents the liquid from crossing the valve; under rotation, the centrifugal pressure difference between the upstream and downstream liquid columns is given by [36,37]:

(1) $$\Delta P=\frac{1}{2}\rho \omega^{2}(R_{2}^{2}-R_{1}^{2}),$$

where $R_{1}$ and $R_{2}$ are the distances from the rotation axis to the centers of the upstream and downstream liquid columns at the valve $R_{2}>R_{1}$; ω=2πn/60 is the angular velocity at rotation speed n (rpm); and ρ is the liquid density. Valve opening occurs when this centrifugal pressure drop exceeds the sum of the burst pressure of the geometric capillary valve ($P_{v}$) and the additional downstream capillary backpressure ($P_{c}$):

(2) $$\Delta P=P_{v}+P_{c},$$

The burst pressure $P_{v}$ was estimated from the Young–Laplace equation for a rectangular cross-section using the channel dimensions and the measured contact angles on PDMS and glass [38]. Combining Equations (1) and (2) yields the threshold angular velocity $\omega_{t}$ and corresponding rotation speed $n_{t}$ for valve opening:

(3) $$\omega_{t}=\sqrt{\frac{2[P_{v}+P_{c}]}{\rho (R_{2}^{2}-R_{1}^{2})}},\;n_{t}=\frac{60}{2\pi }\omega_{\mathrm{th}}\;(\mathrm{rpm}),$$

Using the chip geometric parameters ($R_{2}-R_{1}$ ≈ 15 mm, ρ ≈ 1000 kg·m^−3^, $\gamma_{\mathrm{lg}}$ ≈ 0.06 N·m^−1^), the theoretical threshold rotational speed was calculated to be approximately 1000–1200 rpm. Experimentally, the rotation speed was increased stepwise (800–1500 rpm) while monitoring valve crossing; the first passage occurred at ~1200 rpm, in close agreement with the theoretical prediction. This validates the model and indicates good uniformity in chip fabrication as well as a stable, well-controlled response of the capillary valve.

The centrifugal microfluidic chip, as shown in Figure 3C, was employed for subsequent experiments. The MUF-based fluorescence reaction principle is schematically illustrated in Figure 3D and has been described in Section 2.3. Possible interferences include soil autofluorescence, fluorescence quenching or absorption by humic substances and dissolved organic matter, adsorption of MUF or substrates onto soil particles, light scattering from suspended particles, and pH-dependent variation in MUF fluorescence. These effects were minimized by using soil-specific calibration curves, blank correction, and sedimentation-assisted clarification before fluorescence imaging. To assess the compatibility between the chip architecture and the optical detection system, we evaluated the illumination uniformity over the reaction chamber. With the excitation source and detector fixed, the chip was immobilized on the measurement stage and fluorescence from the same reaction chamber was recorded at multiple rotation angles of the disk. The results show that the measured fluorescence intensity remained essentially constant as the rotation angle changed (Figure 3E), with a maximum relative deviation of <5%. This demonstrates spatially uniform illumination, high optical transparency of the chip materials, and negligible optical perturbation from the chamber geometry or the PDMS–glass interface.

### 3.2. Construction of Calibration Curves

**Figure 4:**
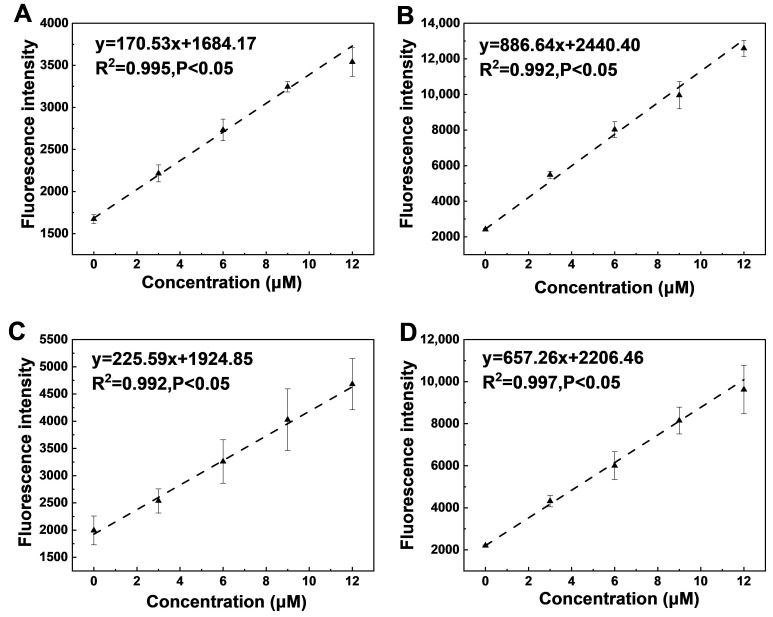
Comparison of calibration curves for different soil samples. (**A**) Red soil calibration curve; (**B**) Saline-alkali soil calibration curve; (**C**) Black soil calibration curve; (**D**) Fluvo-aquic soil calibration curve.

Using the microfluidic-chip platform described above, MUF calibration curves were established for four representative soils, namely red soil, saline–alkali soil, black soil, and fluvo-aquic soil, to evaluate the applicability and accuracy of the microfluidic approach for the quantitative determination of extracellular enzyme activities. As shown in Figure 4, MUF concentration exhibits an excellent linear relationship with fluorescence intensity for all soils (coefficients of determination R^2^ > 0.99). The ImageJ-derived raw fluorescence intensity values used to construct these calibration curves, are provided in Supplementary Table S2. For example, the fitted regressions were y = 170.53x + 1684.17 for red soil (R^2^ = 0.995, *p* < 0.05), and y = 657.26x + 2206.46 for fluvo-aquic soil (R^2^ = 0.997, *p* < 0.05), indicating that a linear model is robust and suitable for subsequent quantification. Based on the slopes, the order is saline–alkali (886.64) > fluvo-aquic (657.26) > black (225.59) > red soil (170.53). The results indicate that, under the same substrate concentration gradient, enzymatic reactions in saline–alkali and fluvo-aquic soils produce stronger fluorescence responsiveness, likely because these matrices contain higher concentrations of soluble base cations and more active surface sites, which enhance enzyme–substrate interactions or stabilize the fluorescent product [39]. By contrast, the lower slopes observed for lateritic and black soils are plausibly attributable to their higher contents of organic matter and iron and aluminum oxides, which can complex or adsorb the analyte, thereby reducing the effective concentration of the target species and attenuating the signal response [40]. Furthermore, intercepts vary substantially among soil types, indicating different matrix contributions to background absorbance and hence baseline fluorescence [41]. Given the pronounced differences in calibration slopes among soil types, a single universal calibration curve is not appropriate. Instead, soil-specific calibration curves, as well as treatment-specific calibration curves where applicable, are required to properly account for matrix effects and to ensure accurate quantification of extracellular enzyme activities.

### 3.3. Enzyme Activities in Different Soils Determined by the Microfluidic Chip

Using the microfluidic-chip method, we quantified eight representative extracellular enzymes, namely BG, CBH, BX, GAL, NAG, NAGA, SF, and AP, in four soil types (red soil, saline-alkali soil, black soil, and fluvo-aquic soil). Activities are reported as substrate conversion rates per gram of sample per hour (nmol g^−1^ h^−1^).

**Figure 5:**
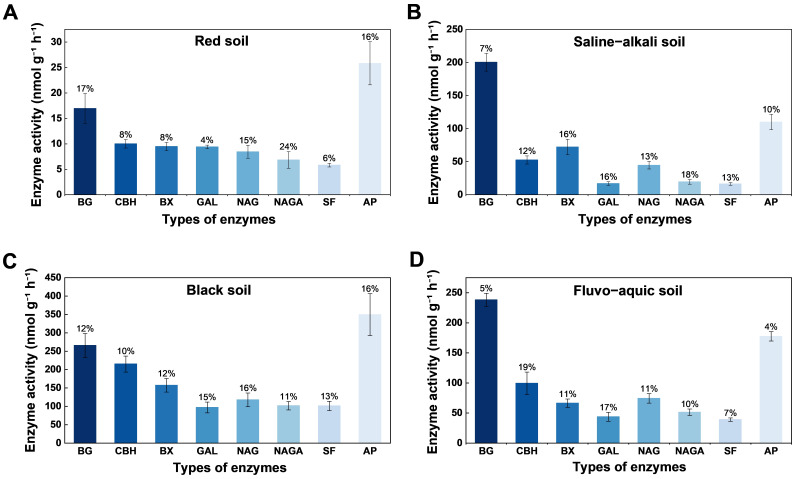
Enzyme activity profiles by soil type: (**A**) Red soil; (**B**) Saline–alkali soil; (**C**) Black soil; (**D**) Fluvo-aquic soil. Data are presented as mean ± SD of replicate measurements; percentages above the bars indicate CVs.

For each enzyme, eight technical replicates were obtained using eight independently prepared microfluidic chips, with the activities of all eight enzymes measured on each chip. For each enzyme, the mean activity, standard deviation and coefficient of variation (CV) were calculated (Figure 5). The bar heights represent activity levels, and the percentages above the bars denote the corresponding CVs, which were used to evaluate the precision and stability of the microfluidic assay.

As shown in Figure 5, when activities are averaged across enzymes, the overall ranking among soils is black soil > fluvo-aquic soil > saline–alkali soil > red soil. The dominant enzyme differs by soil type: BG exhibits the highest activity in saline and fluvo-aquic soils, whereas AP is the most active in red and black soils. This pattern indicates a broadly distributed capacity for carbohydrate hydrolysis and, in the latter two soils, a strong potential for organic-phosphorus mineralization and inorganic-phosphorus release. Activities of NAGA and SF are generally low across soils and are especially lowest in saline–alkali soil (Figure 5B), consistent with suppression under elevated salinity and limited substrate availability [42]. From a methodological perspective, across the four soil types the coefficients of variation (CVs) for the eight enzyme activities ranged from 4–24%, with most CVs < 15%, indicating good repeatability and stability of the microfluidic-chip assay for multiplex determinations. Isolated higher CVs (e.g., NAGA in red soil, 24%; CBH in fluvo-aquic soil, 19%) occurred mainly for weaker signals or in channels more strongly affected by solid particulates, suggesting residual influences of soil microheterogeneity or optical scattering backgrounds. Overall, these low CVs not only demonstrate the internal consistency of the measurements but also provide methodological validation for the suitability and advantages of the microfluidic-chip technique for determining soil extracellular enzyme activities. The platform enables parallel measurement of multiple enzyme classes with minimal sample and reagent consumption, markedly increases analytical throughput, and helps reduce operator-induced error while improving efficiency. In addition, the chip architecture affords tighter control of the reaction environment, rendering enzyme–substrate interactions more controlled and thereby improving measurement precision and repeatability.

Furthermore, the differences in enzyme activities among soil types reveal their underlying ecological functions. The higher activities of carbohydrate-hydrolyzing enzymes and acid phosphatase observed in black and fluvo-aquic soils indicate a strong potential for organic matter decomposition and nutrient mineralization, contributing to the maintenance of soil fertility and the carbon–phosphorus cycles (Figure 5C,D). In contrast, the generally low activities detected in saline–alkali soil suggest that microbial metabolism is constrained by salinity stress and limited substrate availability. Overall, the enzyme activity profiles highlight functional differentiation among soils and serve as biological indicators for evaluating soil quality and guiding sustainable land management. The microfluidic chip platform, with its high throughput and low reagent consumption, offers a promising tool for large-scale monitoring of soil nutrient cycling and environmental management, thereby supporting the sustainable utilization of soil resources.

### 3.4. Validation of the Microfluidic Method for Enzyme Activity Measurement

**Figure 6:**
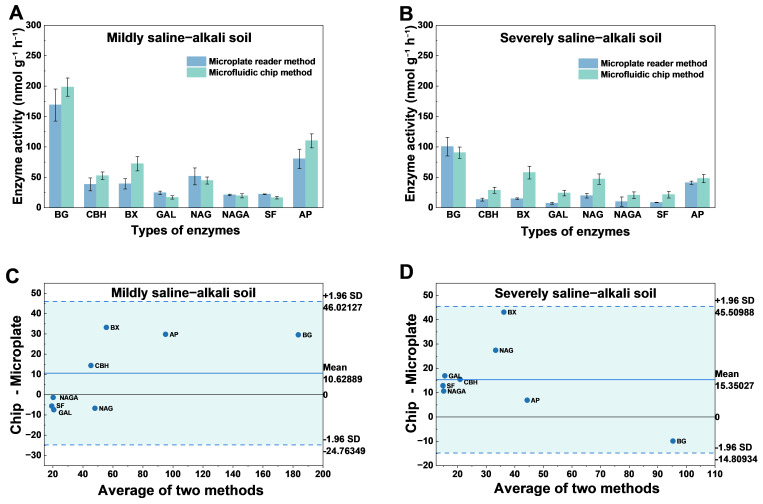
Method comparison and Bland–Altman agreement analysis for enzyme activity measurements in saline–alkali soils. (**A**,**B**) Enzyme activities measured by the microplate reader and microfluidic chip methods in mildly and severely saline–alkali soils, respectively. (**C**,**D**) Bland–Altman plots for mildly and severely saline–alkali soils, respectively. Data in (**A**,**B**) are presented as mean ± SD of replicate measurements. In (**C**,**D**), the solid line indicates the mean difference, and the dashed lines indicate the 95% limits of agreement.

To assess the applicability of the microfluidic-chip method in complex soil matrices, mildly and severely saline–alkali soils were selected as test materials. Activities of eight representative extracellular enzymes—BG, CBH, BX, GAL, NAG, NAGA, SF, and AP—were determined using both the microfluidic chip and a conventional microplate-based fluorometric assay. For each enzyme, eight technical replicates were performed, and the mean activity and standard deviation were calculated. The results under the two soil conditions are shown in Figure 6.

In mildly saline–alkali soil samples, the two methods yielded consistent overall trends, but the microfluidic chip produced higher activities for most enzymes, especially BG, BX, and AP, with activities of approximately 190, 80, and 110 nmol g^−1^ h^−1^, respectively, which were substantially higher than those obtained by the conventional microplate assay. BG, a key enzyme involved in cellulose degradation, exhibited high activity that may reflect a strong microbial demand for organic carbon. The elevated AP activity suggests that phosphorus bioavailability is maintained under saline–alkali conditions, potentially reflecting microbial regulatory responses. Overall, the microfluidic chip demonstrates greater sensitivity to strong signals and smaller variability across replicates (Figure 6A).

In severely saline–alkali soil samples, overall extracellular enzyme activities decline markedly, with BG, GAL, and SF showing pronounced suppression, indicating that strong saline–alkali stress likely impairs both microbial enzyme synthesis and catalytic activity. Notably, even against this low-activity background, the microfluidic chip reliably resolves weak fluorescence signals: enzymes such as BX, NAG, and AP still display comparatively higher activities. By contrast, the microplate fluorometric assay yields lower or more variable readouts for several low-signal enzymes (e.g., GAL, NAGA), revealing limitations in sensitivity under weak-signal conditions (Figure 6B).

To further evaluate the agreement and potential systematic bias between the two methods, Bland–Altman analysis was performed using the paired mean enzyme activity values obtained by the microfluidic chip and microplate reader methods. In mildly saline–alkali soil, the mean difference (chip − microplate) was 10.63 nmol g^−1^ h^−1^, with 95% limits of agreement from −24.76 to 46.02 nmol g^−1^ h^−1^ (Figure 6C). In severely saline–alkali soil, the mean difference was 15.35 nmol g^−1^ h^−1^, with 95% limits of agreement from −14.81 to 45.51 nmol g^−1^ h^−1^ (Figure 6D). All enzyme activity data points were within the limits of agreement, indicating acceptable consistency between the two methods. The positive mean bias suggests that the microfluidic chip generally yielded slightly higher activity values than the microplate reader method, which is consistent with its enhanced fluorescence signal responsiveness in confined micro-reaction chambers.

Overall, the microfluidic chip method showed good agreement with the conventional microplate fluorometric assay while offering advantages in sample economy, reaction homogeneity, fluorescence signal responsiveness, and parallel processing. The confined and sealed micro-reaction chambers can reduce reagent diffusion and evaporative loss, improve enzyme–substrate contact efficiency, and enhance the detection of weak fluorescence signals. In addition, the parallel chip architecture allows multiple enzymes and replicates to be analyzed simultaneously, thereby improving experimental efficiency and reducing manual operation. These results support the applicability of the microfluidic platform for multiplexed soil extracellular enzyme activity measurement in complex saline–alkali soil matrices.

## 4. Conclusions

This study developed and validated a portable, centrifugally driven microfluidic chip for determining soil EEAs. The chip integrates sample aliquoting, reagent metering, mixing, sedimentation-assisted clarification, and fluorescence detection into a unified workflow, enabling parallel measurement of eight enzymes with minimal reagent (≈50 μL) and sample (800 μL) consumption. The system demonstrated high optical uniformity (relative deviation < 5%), reliable capillary-valve liquid control, and excellent calibration linearity (R^2^ > 0.99) across diverse soil matrices. Tests using black, red, fluvo-aquic, and saline–alkali soils showed good reproducibility, with most coefficients of variation below 15%, supporting the suitability of the platform for multiplexed enzyme activity determination in complex soil matrices. Compared with the conventional microplate fluorometric assay, the microfluidic chip showed comparable overall trends and acceptable agreement, as further supported by Bland–Altman analysis. The chip method also more effectively resolved weak fluorescence signals in severely saline–alkali soils, while increasing throughput and reducing manual operation through its parallel architecture. Overall, this portable microfluidic platform provides a rapid, high-throughput, and quantitative approach for soil EEA determination. Its stability, low reagent consumption, and suitability for complex matrices highlight its potential for routine biochemical soil assessment, ecological monitoring, and sustainable agricultural practices. Future developments may integrate AI-driven data analytics and large-scale ecological monitoring frameworks to enhance automation and scalability, further expanding its application in global environmental and agricultural monitoring systems.

## Figures and Tables

**Table 1:** Target extracellular enzymes and corresponding MUF substrates.

No.	Target Extracellular Enzyme	Fluorogenic Substrate
1	β-Glucosidase (BG)	4-MUF-β-d-glucopyranoside
2	N-Acetyl-β-glucosaminidase (NAG)	4-MUF-N-acetyl-β-d-glucosaminide
3	Acid phosphatase (AP)	4-MUF-phosphate
4	Sulfatase (SF)	4-MUF-sulfate
5	β-Xylosidase (BX)	4-MUF-β-d-xylopyranoside
6	Cellobiohydrolase (CBH)	4-MUF-cellobioside
7	β-Galactosidase (GAL)	4-MUF-β-d-galactopyranoside
8	N-Acetyl-β-galactosaminidase (NAGA)	4-MUF-N-acetyl-β-d-galactosaminide

## Data Availability

The original data and contributions presented in this study are included within the article or Supplementary Materials. For further inquiries, please contact the corresponding author.

## Supplementary Materials

The following supporting information can be downloaded at: https://www.mdpi.com/article/10.3390/mi17050599/s1, Table S1: Comparison of representative microfluidic platforms for soil-related applications; Table S2: ImageJ-derived raw fluorescence intensity values for MUF calibration curves in different soil matrices. References [43,44,45,46,47] are cited in the supplementary materials.
