# Supplementary material for: Portable and Point-of-Care Testing Approach for Determining Soil Extracellular Enzyme Activities
Source: Micromachines (Basel). 2026 May 14;17(5):599. doi: 10.3390/mi17050599 (PMC13209134; doi:10.3390/mi17050599)
Supplement: Supplementary file 1 [file micromachines-17-00599-s001.zip › micromachines-4291868-supplementary.pdf]

micromachines

MDPI

Supplementary Materials

Portable and Point-of-Care Testing Approach for Determining Soil Extracellular Enzyme Activities

Xu Han [1,2,3,†], Fangzhou Zhang [2,†], Ruirui Chen [1,4], Weixin Wang [3], Yongjie Yu [5], Zaijiong Yi [3], Jingyi Yang [6], Bo Liu [2], Shilun Feng [2,7], Jun Li [3,*] and Youzhi Feng [1,4,*]

1 State Key Laboratory for Development and Utilization of Forest Food Resources, Nanjing Forestry University, Nanjing 210037, China; hanxu@njfu.edu.cn (X.H.); rrchen@njfu.edu.cn (R.C.)
2 Xiangfu Laboratory, Jiashan 314102, China; fang123zhuan@163.com (F.Z.); liubo@xflab.org.cn (B.L.); shilun.feng@mail.sim.ac.cn (S.F.)
3 Shanghai International Travel Healthcare Center, Shanghai Customs District, Shanghai 200335, China; vivianweiwei5@sina.com (W.W.); 23111010089@m.fudan.edu.cn (Z.Y.)
4 Jiangsu Collaborative Innovation Center for Solid Organic Waste Resource Utilization, Nanjing 210095, China
5 School of Materials Engineering, Changzhou Vocational Institute of Industry Technology, Changzhou 213164, China; yu.yongjie@hotmail.com
6 Nanjing Institute of Geology and Palaeontology, Chinese Academy of Sciences, Nanjing 210008, China; jyyang@nigpas.ac.cn
7 State Key Laboratory of Transducer Technology, Shanghai Institute of Microsystem and Information Technology, Chinese Academy of Sciences, Shanghai 200050, China
* Correspondence: shlijun8150@163.com (J.L.); fyouzhi@hotmail.com (Y.F.)
† These authors contributed equally to this work.

Table S1. Comparison of representative microfluidic platforms for soil-related applications.

References	Detection Principle	Detection Method	Target Analyte	Relative Detection Cost *	Portability	Main Feature or Limitation
Mafla-Endara et al.(2021) [1]	soil-chip visualization	microscopic imaging	Soil microbial communities, fungi, protists, minerals, and pore-space interactions	Medium to high	Medium; chip can be incubated in soil, but imaging requires microscopy	Useful for visualizing in situ soil ecology, but not designed for quantitative biochemical detection.
Arellano-Caicedo et al. (2021) [2]	Artificial soil-pore microstructure combined with fluorescent markers	Fluorescence microscopy	Bacterial/fungal growth and substrate degradation in model soil pores	Medium to high	Low; mainly laboratory-based	Quantifies microbial growth and substrate degradation in artificial pore structures, but uses model systems rather than complex soil suspensions.
Aufrecht et al. (2022) [3]	Rhizosphere-on-a-chip combined with chemical mapping	Liquid micro-junction surface sampling probe mass spectrometry	Root-exuded amino acid hotspots in synthetic rhizosphere pore spaces	High	Low; requires mass spectrometry	Provides spatially resolved rhizosphere chemical analysis, but instrumentation is costly and not field-portable.
Smolka et al. (2016) [4]	Ion separation by capillary electrophoresis	Conductivity measurement	Soil nutrient ions, including NO_3^-, NH_4^+, K^+, and PO_4^{3-}	Medium	High; designed for on-site soil nutrient analysis	Portable and suitable for soil extracts, but focuses on inorganic nutrient ions rather than enzyme activities.

Duford et al. (2013) [5]	Enzyme inhibition-based colorimetric assay	Absorbance detection at 525 nm on centrifugal microfluidic devices	Pesticide residues in vegetable and soil samples	Medium	Medium to high	Integrates extraction, filtration, sedimentation, and detection, but targets pesticide residues rather than endogenous soil enzymes.
Present study	MUF-substrate enzymatic hydrolysis	CCD-based fluorescence imaging	soil extracellular enzymes	Low to medium	High; portable centrifugal microfluidic platform	Enables multiplexed, quantitative soil enzyme activity detection with reduced reagent consumption, good reproducibility, and suitability for complex soil matrices.

* The relative detection cost was qualitatively estimated based on the complexity and accessibility of the required detection equipment. “Low” indicates simple optical or smartphone-compatible readout; “Medium” indicates dedicated but compact optical/electrical detection modules; “High” indicates microscopy- or mass-spectrometry-dependent systems.

Table S2. ImageJ-derived raw fluorescence intensity values for MUF calibration curves in different soil matrices.

Soil Type	MUF Concentration (μM)	Replicate 1	Replicate 2	Replicate 3	Mean	SD
Red soil	0	1634.154	1732.874	1652.888	1673.30533	52.43148
	3	2139.3	2330.296	2176.245	2215.28033	101.30492
	6	2610.215	2718.447	2866.562	2731.74133	128.68955
	9	3187.73	3239.54	3308.458	3245.24267	60.56569
	12	3347.487	3671.293	3600.672	3539.81733	170.26464
Saline-alkali soil	0	2372.364	2400.19	2479.068	2417.20733	55.35005
	3	5526.417	5664.719	5267.129	5486.08833	201.83967
	6	8129.44	8413.677	7538.75	8027.289	446.31876
	9	10508.43	10269.25	9084.02	9953.9	762.77138
	12	12331.52	12335.21	13108.708	12591.81333	447.64771
Black soil	0	1690.467	2131.713	2163.13	1995.103	264.2901
	3	2284.278	2627.889	2696.482	2536.216	220.8641
	6	2806.763	3420.59	3553.067	3260.14	398.1841
	9	3398.009	4491.41	4194.681	4028.033	565.4291
	12	4140.115	5000.159	4902.778	4681.017	470.9589
Fluvo-aquic soil	0	2209.522	2229.387	2177.924	2205.611	25.95346
	3	4174.87	4147.685	4636.107	4319.554	274.4797
	6	6027.561	6656.834	5332.439	6005.611	662.4703
	9	8249.383	8732.395	7463.905	8148.561	640.2269
	12	10647.03	9850.164	8375.048	9624.08	1152.74

References

1. Mafla-Endara, P.M.; Arellano-Caicedo, C.; Aleklett, K.; Pucetaite, M.; Ohlsson, P.; Hammer, E.C. Microfluidic chips provide visual access to in situ soil ecology. *Commun. Biol.* **2021**, *4*, 889. https://doi.org/10.1038/s42003-021-02379-5.
2. Arellano-Caicedo, C.; Ohlsson, P.; Bengtsson, M.; Beech, J.P.; Hammer, E.C. Habitat geometry in artificial microstructure affects bacterial and fungal growth, interactions, and substrate degradation. *Commun. Biol.* **2021**, *4*, 1226. https://doi.org/10.1038/s42003-021-02736-4.
3. Aufrecht, J.; Khalid, M.; Walton, C.L.; Tate, K.; Cahill, J.F.; Retterer, S.T. Hotspots of root-exuded amino acids are created within a rhizosphere-on-a-chip. *Lab Chip* **2022**, *22*, 954–963. https://doi.org/10.1039/d1lc00705j.
4. Smolka, M.; Puchberger-Enengl, D.; Bipoun, M.; Klasa, A.; Kiczkajlo, M.; Śmiechowski, W.; Sowiński, P.; Krutzler, C.; Keplinger, F.; Vellekoop, M.J. A mobile lab-on-a-chip device for on-site soil nutrient analysis. *Precis. Agric.* **2017**, *18*, 152–168. https://doi.org/10.1007/s11119-016-9452-y.

5. Duford, D.A.; Xi, Y.; Salin, E.D. Enzyme inhibition-based determination of pesticide residues in vegetable and soil in centrifugal microfluidic devices. *Anal. Chem.* **2013**, *85*, 7834–7841.

Disclaimer/Publisher's Note: The statements, opinions and data contained in all publications are solely those of the individual author(s) and contributor(s) and not of MDPI and/or the editor(s). MDPI and/or the editor(s) disclaim responsibility for any injury to people or property resulting from any ideas, methods, instructions or products referred to in the content.

https://doi.org/10.3390/mi17050599
